# Non-pharmacological therapies for cancer patients in Portugal and Brazil: an experience report

**DOI:** 10.1590/1980-220X-REEUSP-2023-0091en

**Published:** 2023-09-15

**Authors:** Leonel dos Santos Silva, Ana Filipa Domingues Sousa, Dulce Helena Ferreira de Carvalho, Luciana Puchalski Kalinke

**Affiliations:** 1 Universidade Federal do Paraná, Programa de Pós-graduação em Enfermagem, Curitiba, PR, Brazil.; 2 Instituto Português de Oncologia de Coimbra, Coimbra, Portugal.

**Keywords:** Quality of Life, Complementary Therapies, Integrative Oncology, Oncology Nursing, Neoplasms, Calidad de Vida, Terapias Complementarias, Oncología Integrativa, Enfermería Oncológica, Neoplasias, Qualidade de Vida, Terapias Complementares, Oncologia Integrativa, Enfermagem Oncológica, Neoplasias

## Abstract

**Objective:**

To describe the experience of nurses from a center in Portugal and two in Brazil regarding the use of non-pharmacological therapies in cancer patients.

**Method:**

This is a professional experience report.

**Results:**

In the Portuguese institution, a group of nurses has been performing massage therapy, reflexology, postural teaching, thermotherapy, relaxation, guided imagery, Reiki, music therapy, aromatherapy, among others, for 17 years, with significant results in pain and vital signs with satisfactory perceptions. In Brazil, the clinical application is incipient, clinical studies with auriculotherapy, relaxation with guided imagery and floral therapy are taking place, with significant results for physical symptoms, anxiety, and quality of life improvement.

**Conclusion:**

In both countries, nurses have made efforts to implement non-pharmacological therapies. While in the Portuguese reality they have been formally institutionalized in care, in Brazil the therapies take place with voluntary work and through intervention research. This report can encourage the translation of autonomous practice to clinical studies for proposing evidence of therapies in Integrative Oncology.

## RESUMO

**Objetivo:**

Descrever a experiência dos enfermeiros de um centro em Portugal e dois no Brasil sobre o uso das terapias não farmacológicas em pacientes com câncer.

**Método:**

Trata-se de um relato de experiência profissional.

**Resultados:**

Na instituição portuguesa, há 17 anos um grupo de enfermeiros vem realizando massagem terapêutica, reflexologia, ensinos posturais, termoterapia, relaxamento, imaginação guiada, Reiki, musicoterapia, aromaterapia, entre outras, com resultados significativos na dor, nos sinais vitais e com percepções satisfatórias. Na brasileira, a aplicação clínica é incipiente, ocorrem estudos clínicos com auriculoterapia, relaxamento com imagem guiada e terapia floral, com resultados significativos para sintomas físicos, ansiedade e qualidade de vida.

**Conclusão:**

Em ambos os países, os enfermeiros têm empreendido esforços para implementar as terapias não farmacológicas. Enquanto na realidade portuguesa elas têm sido formalmente institucionalizadas no cuidado, na brasileira as terapias ocorrem de forma voluntária e por meio de pesquisas de intervenção. Esse relato pode fomentar a translação da prática autônoma aos estudos clínicos para a proposição de evidências das terapias na Oncologia Integrativa.

## RESUMEN

**Objetivo:**

Describir la experiencia de enfermeros de un centro de Portugal y dos de Brasil con respecto al uso de terapias no farmacológicas en pacientes oncológicos.

**Método:**

Informe de experiencia profesional.

**Resultados:**

En la institución portuguesa, desde hace 17 años, un grupo de enfermeros realiza masajes terapéuticos, reflexología, enseñanza postural, termoterapia, relajación, imaginería guiada, Reiki, musicoterapia, aromaterapia, entre otros, con resultados significativos en dolor, signos vitales y con percepciones satisfactorias. En Brasil, la aplicación clínica es incipiente, se están realizando estudios clínicos con auriculoterapia, relajación con imágenes guiadas y terapia floral, con resultados significativos para los síntomas físicos, la ansiedad y la calidad de vida.

**Conclusión:**

en ambos países, los enfermeros han hecho esfuerzos para implementar terapias no farmacológicas. Mientras que en la realidad portuguesa han sido formalmente institucionalizados en el cuidado, en Brasil las terapias ocurren de forma voluntaria y por medio de investigación de intervención. Este informe puede incentivar la translación de la práctica autónoma a estudios clínicos para proponer evidencias de las terapias en Oncología Integrativa.

## INTRODUCTION

In the global scenario, with the demographic and epidemiological transition of chronic diseases, health is constantly threatened by the progression of cancer. For the next two decades, an increase of approximately 50% in the incidence of the disease is estimated^(1)^. It is a complex condition, potentially aggravated in low- and middle-income countries due to intangible barriers ranging from diagnosis, conventional therapy, shortage of primary care and palliative care teams to the struggle for quality survival^(2)^.

To face this challenge, numerous cancer patients resort to different potentially therapeutic resources to alleviate a myriad of physical, emotional, and spiritual needs. Furthermore, this complex demand for care promotes changes in health systems and, together with media coverage, promotes non-pharmacological therapies (NPT) as resources to improve health and quality of life, given their positive impact on our system and process of achievement of health outcomes, behavioral, and socioeconomic indicators^(2,3)^.

The NPT is considered “a science-based non-invasive and non-pharmacological human health intervention” whose objective is the prevention, treatment, and cure using different methods, products, programs, and services that are related to biological and psychological mechanisms^(3)^. In this framework, different nomenclatures can represent these therapies: traditional (ancestral knowledge and practices from different cultures prior to the conventional ones), complementary (non-traditional practices used with conventional therapy, although not integrated into the health system), and integrative (concomitant with conventional treatment), with the exception of the alternative (replaces conventional treatment)^(4)^. The absence of universal consensus and the interchangeability of terms are understandable, since the comprehension of conventional or non-conventional treatments differs within regions, countries, and cultures, and may change over time^(5)^.

Frequently, the various NPTs are associated with chronic conditions, although they are not restrictive. Ontologically, they can be classified into five categories: psychological (psychotherapy, health education, and art therapy); physical (manual therapies, physiotherapy, and physical activities); nutritional (food, nutritional and dietary supplements); digital (portable and virtual devices); and health (phytotherapy, aromatherapy, cosmetics, among others)^(3)^.

The implementation of different NPTs has to be based on ethical, relational, and communication skills, supported by research evidence with robust methods and techniques, essential elements to demystify these therapies as less significant. It is necessary to reflect on the provision of care that is less fragmented and more integrated, incorporating evidence of such therapies to promote health and healing^(4)^. These multimodal strategies have been recommended by the World Health Organization since 2002, at different levels for promoting integrative health^(6)^.

Integrative health comprises the use of multimodal (conventional and complementary) approaches, in different combinations, aiming at treating the person as a whole and not just a system of organs, often depending on different providers, institutions, and professionals^(4)^. It is about reaffirming the relationship between the professional and the patient and is person-centered, informed by evidence; it makes use of therapeutic approaches and lifestyle modifications. Patients seek professionals who can understand the oneness of mind, body, and spirit to create their own health balance. This will require a restructuring of education, research, and model of care, a shared view to address the unique individuals’ health needs^(4)^.

Adjusted to the integrative and holistic concept of health, in Integrative Oncology (IO), person-centered care prioritizes safety and the best available evidence in the provision of interventions for the mind and body, natural products, and the lifestyle of different traditions, concomitantly to conventional treatment. In its turn, proper integration of NPTs may result in an improved amount and quality of life for individuals with cancer^(2)^. Therefore, it is essential that integrated oncology care teams are effective in assertive communication when defining realistic expectations, based on critical and scientific analyses of medical and therapeutic conditions, with the objectives of conventional treatment and NPTs.

This study is justified by the conceptual proposition of IO, which is not restricted to curing or prolonging survival, but also to alleviating symptoms, improving quality of life and physical, emotional and/or spiritual well-being with the use of different strategies and holistic view of care, highlighting the increasing prevalence of NPTs and resulting in the need to implement integrated health services^(5,7)^. Therefore, the question is: what is the experience of a group of Portuguese and Brazilian nurses in the implementation of non-pharmacological therapies in the care of patients with cancer? The objective was to describe the experience of nurses from a center in Portugal and two in Brazil regarding the use of non-pharmacological therapies in cancer patients.

## METHOD

### Design of Study

This is a descriptive professional experience report on the use of non-pharmacological therapies by nurses in three hospitals specialized in cancer treatment, one in Portugal and two in southern Brazil. This type of study describes a fact or professional/institutional experience about a specific situation that can be inspiring for the implementation of praxis and encourage the development of new objects of investigation. There is still no guideline to direct the writing^(8)^.

### Local

The experience described was lived in three tertiary care units. In Portugal, this is a reference hospital in the diagnosis and treatment of oncological diseases, which is part of the care network of the National Health Service. It has an innovative structure that concentrates different technological and human resources, and is located in the central region of Coimbra, which has an estimated population of two million inhabitants. It is one of the institutions responsible for building and executing the national oncology policy, engaged in care, professional training, and research development. It assists cancer patients in their different diagnostic and therapeutic, clinical and surgical needs, with its various multidisciplinary specialties, with emphasis on pain treatment units, palliative care, and hospice.

In Brazil, these are two high-complexity public hospital complexes, located in the capital of the state of Paraná, in the southern region of the country, here called A and B.

Hospital complex A is linked to a public and federal university, considered the largest in the state, has teams in different specialties, and encourages multidisciplinary residency. It provides outpatient and inpatient care for cancer patients, in chemotherapy, clinical and surgical treatment, is nationally and internationally recognized as a center of excellence in bone marrow transplantation. Institution B is philanthropic and specialized in integrated care, teaching and research from cancer diagnosis to treatment, predominantly serving patients from the public health system. It brings together different clinical and surgical specialties, as well as multiprofessional residency training in Oncology. It is responsible for a large number of surgeries, chemotherapy, is a pioneer in radiotherapy and brachytherapy, as well as bone marrow transplantation services, palliative care, being the only hospice with care provided by the public system in the southern region of the country.

### Ethical Aspects

This experience report reflects the impressions and describes the professional activities developed by the authors inserted in their respective teams, as well as some organizational aspects of the work process. In view of the above, despite the waiver of submission for approval by the ethics committee, since no form of data collection involving human participants was carried out, the current ethical precepts were observed.

## RESULTS

### Portugal Experience

The oncological hospital located in the central region of Portugal began its care activities in 1923. From 2005 onwards, after a request from the Ministry of Health, a group of nurses was mobilized to propose an integrative project to promote well-being using NPTs. These nurses joined different specific training courses and were allocated to the pain unit, according to the guidelines of the institutional direction. In the country, this oncology hospital was a pioneer in incorporating a team of nurses into a pain unit that would provide autonomous and integrative care. It was implemented in 2011 and called “NPT Group”.

Initially, the hospital provided basic training (relaxation techniques, massages and thermotherapy) for the beginning of care, which continues to be made available today. After that, some nurses invested in extra-hospital academic training, a fact that enriched and valued the group with new therapies and knowledge that is shared with the other members. Currently, the group is articulated with the institution’s continuing education and internally promotes training and certification courses (Reiki level I, music therapy, aromatherapy and guided imagery) for new entrants, aiming at training nurses who will work in the NPT Group.

In 2022, on the international nurses day and fight against pain day, events to publicize the work carried out by the group were held and NPT sessions were made available so that the internal community could notice its advantages. In addition, there was another event about therapies aimed at the external community, directed to informal caregivers. Currently (2023), the group is made up of eight nurses from different clinical, surgical, or outpatient units. Every week, two professionals are transferred from their respective care units to work with the NPTs and they do not receive additional remuneration for this activity, since on the day they work in the group they do not carry out concomitant activity in their respective care unit.

For a nurse from the institution to join the NPT group, the professional’s interest, the existence of a vacancy, and the candidate having participated in the available training courses are required. Once these requirements are met, the group leaders carry out an analysis of the curriculum (history at the institution, professional experiences, and additional training) and of the candidate’s letter of motivation (founded justification on interest in working with therapies) regarding practical skills based on primary training previously made available. Approved in the selection stages, the nurses integrate the group and participate in the training and certification courses, and then they will be able to work with the different therapies.

Among the objectives of the TNF group are: to decrease anxiety and optimize quality of life, provide relief and increase the level of pain tolerance, enable the patient to develop pain and anxiety control strategies, reduce the use of pharmacological strategies, enhance the patient’s self-esteem and autonomy with a focus on the biopsychosocial aspects of pain^(9)^. An information guide was developed by the NPT group for patients and professionals to clarify the objectives of the interventions. This information was posted on the intranet and on the institutional website, as disclosure is understood as a construct of care, providing excellent and comprehensive care.

Initially, patients were predominantly referred by anesthesiologists, a specialty that performs pharmacological management and identifies patients with uncontrolled pain, who can benefit from NPT. In the last three years, just over 50% of patients have been referred by other different clinical and surgical specialties, a fact that is justified by the recognition of the NPT group before the institutional community, by the large number of health professionals who know about the group’s existence, the internal validity of the benefits of the therapies and the functioning.

Despite the lack of a flowchart with well-defined criteria, patients referred to the NPT group almost exclusively have chronic pain. In cases of acute pain, admissions occur after pharmacological control. It should be noted that regardless of the pain score, patients can be referred, including those with no pain and prevalence of anxious symptoms. Nurses may refer patients for NPT intervention, as long as it is a shared decision with the medical team.

Registration was one of the main concerns of the group, from admission conditions up to their progression to NPT. The taxonomy used to standardize and systematize with a standardized language is the International Classification for Nursing Practice (ICNP), carried out in electronic medical records. In addition, prior to the beginning of the therapy sessions, the patient is asked to sign the informed consent form after a detailed explanation of the therapies, their objectives, planning, the concomitant integrative purpose of the conventional treatment and the deconstruction of ideas that such therapies are less effective or derived from religious practices.

Following patient admission, eight sessions of NPT are planned, applied weekly, lasting 45 minutes. Later, if the patient decides to receive new sessions, it is necessary to wait in a queue due to the demand and considering that there are only eight nurses in the group. Among the NPTs available, there are: therapeutic massage, application of heat/cold, relaxation, Reiki, music therapy, aromatherapy, reflexology, guided imagery, postural teachings, among others. The institution pays for the necessary supplies, such as sweet almond oil, gloves, sheets, towels, heaters, caps, etc.

Prior to the beginning of the session, history taking and evaluation of pain and/or psychometric measurement of anxiety, measurement of vital signs pre and post-intervention are carried out, and subsequent steps and follow-up visits are discussed, as well as the importance of a patient companion during the intervention. After evaluation, the nurse has autonomy to decide and dialogue with the patient, based on his/her clinical judgment and the therapeutic plan, that is, he can apply one or more interventions during the same session, based on his/her training, positive results and preferences of the patient. An institutional protocol for the application of NPT sessions has not yet been validated, precisely because of the understanding that the essence of integrative care is to serve the patient in a holistic way and focusing on their individual needs.

Based on the latest records, approximately 200 annual sessions were accounted for, which were suspended from March 2020 to September 2021 due to COVID-19. Positive results are identified before the eighth session. In general, virtually all participants show improvement in pain intensity and report relaxation at the end of the sessions. Quantitatively, positive results are observed in vital signs monitored after NPT. Qualitatively, we welcome reports of different satisfactory perceptions after the sessions held.

The group’s professionals hold periodic meetings to discuss clinical cases, exchange experiences, and share available scientific evidence on the NPT they apply, in addition to the annual scientific event, and there are plans to develop clinical research. In the perception of the NPT group, after 17 years of activity, the work developed is institutionally recognized and the service is considered a reference and skeptical peers at some point recognize the benefits of therapies. The medical team also recognizes the added value of therapies, as they are often surprised by positive reports after sessions.

### Experience from Brazil

In the institutional reality of the two institutions in southern Brazil that were reported in the present study, it is observed that the implementation of NPT in the care practice of patients with cancer is incipient. The nurses who practice them do it in an isolated and non-institutionalized way, that is, they apply it because they observed in previous studies that the patients showed an improvement, that the practices were easy to be taught, the cost was zero, or low, and the professional had autonomy to perform them. In the institutions, they do not support the training of professionals for the use of NPT, and the nurses who use the therapies have received external and independent training.

Therefore, in this article, the experience that will be described is based on research carried out in both institutions, linked to the Graduate Programs in Nursing at the Universidade Federal do Paraná. It was based on these surveys, at both institutions, that many were able to learn about or become familiar with NPT, which until then were only present in the literature, allowing demystification of their use and projection of their provision to cancer patients.

The first study was developed in the professional master’s degree at institution A, using the technique of auriculotherapy with crystal spheres that was applied during chemotherapy treatment for breast cancer. The results showed that, in the intervention group, women had a better overall quality of life, with statistical significance on the nausea-vomiting scale and breast discomfort, in addition to clinical improvement in other physical and emotional symptoms, although without significance^(10)^. After the completion of this study, more than 50% of the outpatient nurses sought external training to apply the intervention to patients, but this did not last due to lack of institutional support.

In institution B, in a dissertation in progress, the technique of auriculotherapy was also researched, but applied to laser in patients with breast cancer on palliative therapy, with the objective of evaluating the outcome in quality of life and anxiety. This is a clinical study with 123 women randomized into three groups: control, pseudo-control, and intervention, for 10 weeks. Previous results (not yet published) have shown improvement in anxiety, fatigue, and some domains of quality of life.

At institution A, a master’s research with a quasi-experimental design used guided imagery and progressive muscle relaxation with the use of virtual reality glasses in patients undergoing hematopoietic stem cell transplantation. The intervention applied for four weeks consisted of the reproduction of a narration that guided the patient to relaxation, projection of images and sounds of nature in 360º with instrumental music, lasting approximately ten minutes. It should be noted that most of the unit’s professionals were trained and they were involved in performing the technique on patients. Positive outcomes were observed in vital signs, with clinically statistical significance before and after the intervention in temperature, oxygen saturation, respiratory and heart rate^(11)^.

At institution B, research carried out in the professional master’s program also evaluated the effect of image-guided virtual reality on anxiety and quality of life of women with cervical cancer undergoing radiochemotherapy. In this randomized clinical trial, participants (n = 52) were followed for 12 weeks during combined chemotherapy and radiotherapy. The results showed that the patients who received the intervention had a statistically significant decrease in anxiety and obtained clinical improvement in the physical, emotional, global quality of life domains and in the domain in which the changes and specific concerns of this type of cancer are evaluated^(12)^. It should be noted that after the development of this study, some nurses applied the intervention in isolation, but it was discontinued due to the lack of institutional support.

Another study is in the data collection phase to combine the effect of Bach Flower Therapy in patients with advanced cancer. The therapy outcomes versus placebo will be evaluated in the construct of hope and domains of quality of life. Even because it is a randomized clinical trial with double blinding and without previous results, the researchers experience different reports of improvement in emotional symptoms, sleep, mood, worry, tranquility and others.

## DISCUSSION

In the different health systems, there is a growing movement for the implementation of integrative health, either to meet the demands of cancer patients or to reduce costs. However, it is an activity permeated by challenges in what regards organizational culture, funding, patient experience, adequacy of physical spaces, team training and accreditation, although the greatest complexity is transposing the disease-centered model into the health and well-being model, as well as mitigating communication barriers about or between NPTs and conventional treatment^(13)^.

In Portugal, regulation of the use of NPTs began in 2003 and was consolidated with the Health Bases Law in 2019, still with no broad universal coverage. Despite this, therapies are not available in institutions linked to the public health system, a fact that restricts their use to the urban population, with higher educational and economic conditions; this highlights weaknesses in accessibility and availability, which requires urgent action to ensure universal coverage. It is important to recognize in advance that the regulatory process in the country has promoted safety for patients and professionals with regard to the use of therapies, training, legal and deontological support^(14)^.

Only massage techniques have been authorized by the Portuguese Nursing Council (*Ordem dos Enfermeiros Portugueses)* since 2016, which can be included in the therapeutic plan according to the evaluation results and available resources^(15)^. It should be noted that the country has undergraduation courses in Acupuncture, Phytotherapy, Homeopathy, Traditional Chinese Medicine, Naturopathy, Osteopathy, and Chiropractic.

Brazil has had a National Policy on Integrative and Complementary Practices (*PNPIC*) inserted in the Brazilian Public Health System (*SUS*) since 2006 and is a reference in the provision of NPTs in Primary Health Care (78%), but they are not routinely prescribed to oncology patients, given its low availability in high complexity care (4%)^(16)^. The Federal Nursing Council (COFEN) has supported, since 1997, through resolution No. 197/1997^(17)^, Brazilian professionals practice in the different therapeutic modalities contained in the *PNPIC;* however, this resolution was revoked by resolution No. 500/2015^(18)^ and currently the 581/2018^(19)^ recognizes these therapies as a nursing specialty, upon completion of a graduate certificate course, the legally instituted title to work in public or private services, as well as compliance with ethical precepts, standards, and institutional protocols in different areas and levels of care.

The Brazilian national health survey highlighted a prevalence (5.2%) of NPTs use in 2019, with emphasis on medicinal plants and herbal medicines (3%), acupuncture (1.4%), homeopathy (0.9%), meditation (0.7%) and yoga (0.4%) as the most used in the northern region of the country by women and the elderly. Other aspects that reflect this heterogeneity of Brazilian regions is that they are more used by people with higher income, higher education, and private health insurance, who benefit from therapies that depend on professionals and specialized supplies (acupuncture and homeopathy). The population with less economic and educational conditions uses plants and herbal medicines due to easy access and predominantly without professional guidance^(20)^.

Given the Brazilian territorial extension and the quanti- qualitative disparities of health services, the implementation of IO, although far from ideal, has been increasing in the high complexity of the best private hospitals and in some public institutions specialized in cancer in the states of São Paulo and Rio de Janeiro. Among the most used NPTs, there are: meditative techniques and massage, herbal medicines and anthroposophy^(21)^. In an observational study carried out at a university hospital in the capital of Minas Gerais, NPTs were used by patients (77.1%) undergoing chemotherapy, although few (7.4%) were funded by the *SUS*, focused on spiritual practices (50%) and phytotherapy (26.6%), with benefits in general well-being, tranquility, sleep improvement, pain control, and spiritual comfort^(22)^.

In developed countries, integrative health is an emerging area that is progressing rapidly in the translation of research, practice, education, and policies. Nations that ignore such importance will do so at economic and health expense^(23)^. The reported prevalence of the use of different NPTs in the last 12 months has ranged from 24 to 71.3%, data indicated by a systematic review of 40 primary studies from 14 developed countries^(24)^. An example of this is that, in European countries, the use of NPTs in cancer patients was estimated at 37% with an exponential increase (69%) of IO services in the public health sector^(2)^.

Approximately 60% of French cancer patients make free use of a range of NPTs in different services, with emphasis on homeopathy, nutritional counseling, acupuncture, auriculotherapy, psychotherapies, mind-body therapies, aromatherapy, and aesthetic cancer support^(7)^. In France, an open and multidisciplinary university platform brings researchers together in a collaborative way, aiming at synthesizing evidence of interventional research with NPTs, developing a taxonomy and ontology that converges professional experience and artificial intelligence with a specific search engine for therapies in cancer prevention and treatment^(3)^. In Brazil, the Brazilian Academic Consortium of Integrative Health is responsible for bringing together and promoting the collaboration of researchers in a network that generate guidelines based on systematic reviews and clinical evidence of the different NPTs to meet the challenges of global health with a working committee on IO^(25)^.

In the United States, the global prevalence of NPT use is 35% and, despite the divergences between drug interactions and phytotherapy, its use is increasing in IO. Although there are limitations, there are many evidence-based guidelines, notably for acupuncture, massage therapy and relaxation techniques, homeopathy, herbal medicine, and natural supplements^(2,24)^. In the North American health system, the provision of NPTs is facilitated, although they have not yet been implemented in the hegemonic conventional system due to barriers related to evidence^(5)^.

China envisions an example of success with its secular Traditional Chinese Medicine (TCM) used by 80% of cancer patients in an integrated way, especially phytotherapy, *tai chi/qi gong,* and acupuncture. NPTs are safely provided in several public hospitals and the government invests in research. Despite advances, the country and surroundings still face challenges in providing comprehensive and equitable services, they are unaware of the side effects of all herbs, they are faced with logistical difficulties, among others^(2)^. TCM is implemented at all levels of health care and, despite the high number of clinical studies, it still lacks better evidence, collaboration of professionals who work in an individualized and classic way, concomitantly with conventional medicine, to develop a guideline for clinical practice in the context of integrative health^(5)^.

In Latin America, it is estimated that (50% to 90%) cancer patients use NPTs, even with incipient evidence. In some countries (Argentina, Chile and Brazil), they are rarely performed in hospitals, but among the most used are: natural products, mind and body practices (meditation, yoga, tai chi, acupuncture, massage, music therapy, dances, mandalas, and horticulture)^(2)^.

An urgent reflection is required on integrative health and its potential benefits in the complex health-disease-care process, intertwined by the dimensions of emotion, intuition and sensitivity, which are not always tangible in robust clinical research methods. Giving visibility to intercultural knowledge and practices (knowledge ecology) paves the way for the decolonization of health, allows for an expanded and intercultural experience of the concept of care. Moreover, associated with the distressing issues regarding insufficient funding, training and research with the use of different NPTs, it calls for reflection and action in IO, as well as in global health^(26)^.

The Portuguese experience is motivating and needs to be closer to research; that of Brazilians, however, shall go through the translation of research into clinical practice, but for both, the implementation of NPTs is permeated by many challenges. There is still a need to restructure the ways in which patients are referred: on a case-by-case basis, based on a flexible protocol or on specific conditions. Recent research points out that, in the global scenario, a major advance that incorporates and regulates professionals with NPT expertise, develops protocols, expands the provision in cases of limited or potentially toxic conventional treatments, improves interprofessional communication, records the effects, expands the financing of therapies in different health systems and training of teams^(27,28)^ is of utmost importance.

The success of the process depends on the articulation with different actors: institution (to promote collaboration and education among care teams, as well as engagement of professional societies), patient (to engage individuals and families in clear communication about the intervention, effectiveness, and goals), health system (to identify barriers and facilitators to promote interprofessional collaboration), community (to promote patients’ rights defense groups), financial (to align therapies with health system objectives and to measure the socioeconomic impact of therapies with the burden of patients’ symptoms), and regulatory (to overcome normative and legal obstacles)^(5)^.

As a limitation of this study, the data presented here cannot be generalized, as they result from the narrative and reflections of the professional practice of a limited group of nurses who participated in the broad process in different institutions, since new perceptions and interpretations could be dichotomously presented if described by all group members. Hopefully, this study will motivate and implement the use of NPTs in IO.

## CONCLUSION

The translation of knowledge developed about non-pharmacological therapies has promoted global transformations in the health-disease-care process, mainly with regard to equitable supply, formulation of evidence, public policies, and transposition of the hegemony of conventional therapy to a vision of Integrative Health in Oncology, given the epidemiological transition experienced.

In the complexity of this process, the experiences of different institutions and health systems stand out, but with a close vernacular relationship. In Portugal, nurses made efforts to implement different therapies that were formally instituted and supported by the hospital, even without a clear level of evidence or wide professional regulation that authorizes their use by the profession, but which in clinical practice have shown results in health indicators. In Brazil, in spite of having legislation that recognizes therapies by the *SUS*, of the broad legal support that authorizes the work of nurses in this area, we still transit in the voluntary development of intervention research, without institutional support for the integralization of therapies in care and still timid in the implementation of therapies in Integrative Oncology.

Aware that there is a long path to be taken by professionals from both realities, but regardless of the route, the nurse is present at different levels of care, acting in clinical practice, research or public policy management, and must make efforts to implement the vision of Integrative Health and base their actions on the best evidence aimed at cancer patients’ quality of life, besides promoting their professional autonomy.

## References

[1] Ferlay J, Laversanne M, Ervik M, Colombet M, Mery L, Piñeros M (2023). Global Cancer Observatory: Cancer Tomorrow [Internet]..

[2] Mao JJ, Pillai GG, Andrade CJ, Ligibel JA, Basu P, Cohen L (2022). Integrative oncology: addressing the global challenges of cancer prevention and treatment.. CA Cancer J Clin..

[3] Plateforme CEPS. (2023). Plataforma universitária Colaborativa para a Avaliação de Programas de Prevenção e Cuidados de Apoio [Internet]..

[4] Rakel D, Minichiello V (2023). Integrative Medicine.

[5] Chung VC, Ho FF, Lao L, Liu J, Lee MS, Chan KW (2023). Implementation science in traditional, complementary and integrative medicine: an overview of experiences from China and the United States.. Phytomedicine..

[6] World Health Organization. (2019). WHO Global report on traditional and complementary medicine 2019 [Internet]..

[7] Toledano A, Rao S, Frenkel M, Rossi E, Bagot JL, Theunissen I (2021). Integrative oncology: an international perspective from six countries.. Integr Cancer Ther..

[8] Casarin ST, Porto AR (2021). Relato de Experiência e Estudo de Caso: algumas considerações.. J Nurs Heal [Internet]..

[9] Rogers AH, Farris SG (2022). A meta-analysis of the associations of elements of the fear-avoidance model of chronic pain with, negative affect, depression, anxiety, pain-related disability and pain intensity.. Eur J Pain.

[10] Vallim ETA, Marques ACB, Coelho RCFP, Guimarães PRB, Felix JVC, Kalinke LP (2019). Auricular acupressure in the quality of life of women with breast cancer: a randomized clinical trial.. Rev Esc Enferm USP..

[11] Silva LAA, Guimarães PRB, Marques ACB, Marcondes L, Barbosa CS, Costa PCP (2022). Effects of guided imagery relaxation in hematopoietic stem-cell transplantation patients: a quasi-experimental study.. Rev Bras Enferm..

[12] Santana EO, Marcondes LM, Silva LAA, Sawada NO, Rosa LM, Kalinke LP (2023). Rev Cuid.

[13] Hermanson S, Pujari A, Williams B, Blackmore C, Kaplan G (2021). Health Care.

[14] Amaral P, Fronteira I (2021). Regulation of non-conventional therapies in Portugal: lessons learnt for strengthening human resources in health.. Hum Resour Health..

[15] Portugal. Ordem dos Enfermeiros. (2016). Massagem Terapêutica na Consulta de Enfermagem na Unidade de Dor Crónica [Internet]..

[16] Brasil. Ministério da Saúde. (2023). Práticas Integrativas e Complementares [Internet]..

[17] Conselho Federal de Enfermagem – Cofen. (2023). Resolução nº 197/1997 [Internet]..

[18] Conselho Federal de Enfermagem – Cofen. (2023). Resolução nº 500/2015 [Internet]..

[19] Conselho Federal de Enfermagem – Cofen. (2023). Resolução nº 581/2018 [Internet]..

[20] Boccolini PMM, Boclin KLS, Sousa IMC, Boccolini CS (2022). Prevalence of complementary and alternative medicine use in Brazil: results of the National Health Survey, 2019.. BMC Complement Med Ther..

[21] Brasil. Ministério da Saúde. Secretaria de Atenção Primária à Saúde. (2018). Práticas integrativas auxiliam no tratamento contra o câncer [Internet]..

[22] Gurgel IO, Sá PM, Elaine P, Leal M, Reis IA, Mattia AL (2019). Prevalência de práticas integrativas e complementares em pacientes submetidos à quimioterapia antineoplásica.. Cogitare Enferm..

[23] Seetharaman M, Krishnan G, Schneider RH (2021). The future of medicine: frontiers in integrative health and medicine.. Med..

[24] Lee EL, Richards N, Harrison J, Barnes J (2022). Prevalence of use of traditional, complementary and alternative medicine by the general population: a systematic review of national studies published from 2010 to 2019.. Drug Saf..

[25] (2023). Consórcio Acadêmico Brasileiro de Saúde Integrativa – CABSIn [Internet]..

[26] Guimarães MB, Nunes JA, Velloso M, Bezerra A, Sousa IM (2020). Integrative and complementary practices in the health field: towards a decolonization of knowledge and practices.. Saude Soc..

[27] Chung VCH, Ho LTF, Leung TH, Wong CHL (2021). Designing delivery models of traditional and complementary medicine services: a review of international experiences.. Br Med Bull..

[28] Hunter J, Majd I, Kowalski M, Harnett JE (2021). Interprofessional communication – a call for more education to ensure cultural competency in the context of traditional, complementary, and integrative medicine.. Glob Adv Health Med..

